# Pervasive structural racism in environmental epidemiology

**DOI:** 10.1186/s12940-021-00801-3

**Published:** 2021-11-17

**Authors:** Melissa J. Perry, Suzanne Arrington, Marlaina S. Freisthler, Ifeoma N. Ibe, Nathan L. McCray, Laura M. Neumann, Patrick Tajanlangit, Brenda M. Trejo Rosas

**Affiliations:** Department of Environmental and Occupational Health, Milken Institute School of Public Health, The George Washington University, 950 New Hampshire Ave NW Suite 400, Washington, DC, 20052 USA

**Keywords:** Environmental health, Environmental epidemiology, Environmental justice, Racism, Structural racism, Male reproductive health, Solutions

## Abstract

**Background:**

Epistemological biases in environmental epidemiology prevent the full understanding of how racism’s societal impacts directly influence health outcomes. With the ability to focus on “place” and the totality of environmental exposures, environmental epidemiologists have an important opportunity to advance the field by proactively investigating the structural racist forces that drive disparities in health.

**Objective:**

This commentary illustrates how environmental epidemiology has ignored racism for too long. Some examples from environmental health and male infertility are used to illustrate how failing to address racism neglects the health of entire populations.

**Discussion:**

While research on environmental justice has attended to the structural sources of environmental racism, this work has not been fully integrated into the mainstream of environmental epidemiology. Epidemiology’s dominant paradigm that reduces race to a mere data point avoids the social dimensions of health and thus fails to improve population health for all. Failing to include populations who are Black, Indigenous, and people of color (BIPOC) in health research means researchers actually know very little about the effect of environmental contaminants on a range of population health outcomes. This commentary offers different practical solutions, such as naming racism in research, including BIPOC in leadership positions, mandating requirements for discussing “race”, conducting far more holistic analyses, increasing community participation in research, and improving racism training, to address the myriad of ways in which structural racism permeates environmental epidemiology questions, methods, results and impacts.

## Introduction

Currently, epistemological biases in environmental epidemiology prevent the full understanding of how racism’s societal impacts directly influence health outcomes. The field continues to parameterize conditions of communities of color [1] without recognizing that these social forces are in fact root causes of many disease etiologies. If public health researchers seek to achieve health equity for persons of all backgrounds, the impact of racism on health outcomes needs to be acknowledged, quantified, and addressed. This will require advancing paradigms that identify how racism affects both population health and the health research enterprise. To address racism in public health, our own racist structures need to be examined and dismantled.

Leading medical and public health institutions have long recognized that racism perpetuates health disparities [2, 3]; numerous calls have been made over the last several decades to reform how race and racism are examined in epidemiology [4–7]. These calls have largely been ignored [8, 9], populations of color continue to be underrepresented in research, and structural factors that drive racial disparities endure. The tendency to attribute racial health differences to biology or genetics persists in studies published in today’s top journals [10, 11] and were seen in early explanations for the disproportionate impact of COVID-19 on Black, Indigenous, and people of color (BIPOC) communities [12, 13].

Structural racism, consisting of societal forces, policies and institutions that interact to produce and maintain racial and ethnic inequities [14] is insidious. In addition to public health, it permeates the criminal justice system, labor, credit and housing markets, academia, and the health care sector [7]. It is at the core of why vast racial health disparities persist despite promises from the U.S. Department of Health and Human Services’ *Healthy People* goals, announced in 2000, to eliminate them by 2010 [15]. Today, the life expectancy of Black men is 8 years lower than women of other races, 6 years lower than Black women, and lower than all groups of men [16–18]. The 82.7 year life expectancy of U.S. Hispanics’ is higher than Non-Hispanic Whites’ (80.6 years) [19], however disaggregating these data reveal that U.S.-born Black, American Indian/Alaska Native (AI/AN), Other, or Multiple Race Hispanic adults have considerably lower life expectancy than U.S.-born White Hispanic adults [20]. AI/AN men and women have an overall life expectancy of 78.4 years [17], whereas life expectancy among Non-Hispanic AI/AN in or adjacent to federally-recognized tribal areas, a demographic that is less subject to racial misclassification, is markedly lower, at 71.1 years [21]. Differences in health and health outcomes by race have been attributed in part to language and cultural barriers, lack of access to care, and lack of health insurance [22] and can persist despite accounting for socioeconomic status [7]. That many communities of color are burdened by adverse health outcomes such as cardiovascular disease, infant mortality, diabetes, and certain cancers at much higher rates than Whites has been documented for decades but these disparities have not been fully explained let alone eliminated [7, 18].

It has long been recognized that the social construct of race is inextricably linked with environment [23] and that geography is central to the process of racialization [24, 25]. Using geography to socially segment people is a fundamental implement of racism [26]. With the ability to focus on “place” and the totality of environmental exposures, environmental epidemiologists have an important opportunity to advance the field by proactively investigating the structural racist forces that drive disparities in health. Place matters; structural racism cannot be dismantled until the full effect of place on health is realized, and environmental health is inherently positioned to interrogate it. Therefore, the evaluation and dismantling of structural racism should be an environmental health research imperative.

## Objective

The objective of this commentary is to provide a detailed evaluation of how structural racism affects environmental health and epidemiology, reviewing how it impacts public health education and its research pipeline; research subject matter, funding, methodology, and publication; and the translation of research into policy and practice. We use a few examples from our own research in male infertility to illustrate how failing to include diverse populations in epidemiologic research perpetuates systemic disparities in health. These examples are followed by recommended solutions to remove racist assumptions from public health research.

## Discussion

### The problem in environmental health research

For over four decades environmental health scholars have documented how structural racism drives disproportionate siting of industrial pollution facilities near communities of color [27–30], however this dark legacy continues. Nationally, Black children are still more exposed to lead than children of other racial groups [31]. Native Americans bear increased environmental exposure burden from the impact of the mining industry in many western states [32]. Asian, Hispanic, and Black Americans bear a disproportionately high air pollution burden and are more likely to experience environmental exposures through drinking water compared to White Americans [33, 34]. The combination of these and other disproportionate exposures have contributed to higher body burdens of many toxins in communities of color, including lead and other heavy metals, polychlorinated biphenyls, particulate matter, and phthalates [35]. Epidemiologic and toxicologic data have long documented the adverse impact of these toxins on neurological, endocrine, respiratory, and cardiovascular health, and multiple survey studies have reported that BIPOC populations are concerned about their high exposures to environmental pollution [36–38].

Environmental justice (EJ) literature is replete with examples depicting the burden of structural environmental racism on the exposures and health of people of color [33, 39–49]. Morello-Frosch and colleagues [46] found that Southern California Asian, African American and Latino residents were significantly more likely to live near toxic facilities and had higher lifetime cancer risks than White residents. Longitudinal analyses revealed that these facilities were almost always sited in existing communities of color, disproving a “minority move in” hypothesis [46]. Tessum and colleagues [47] found that nationally, Blacks and Hispanics had a 56 and 63% average PM 2.5 pollution burden respectively, relative to their consumption of goods, whereas Whites had a minus 17% average PM 2.5 exposure burden relative to their consumption. Other leading environmental health researchers have documented the impact of neighborhood-based exposures on psychosocial stress, an important mediator for many health outcomes [49] and the contribution of urban revitalization and gentrification to environmental inequity [48].

Unfortunately, these and other EJ studies are the exceptions to the rule within the broader field of environmental health and epidemiology, possibly because EJ and environmental health originate from different movements. Though environmental health is a long and storied discipline, modern environmental health developed out of widespread environmental concern following the publication of Rachel Carson’s *Silent Spring* in 1962, which ushered in a new era of Western environmentalism that recognized critical connections between the environment and human health [50]. The environmental justice movement on the other hand originated out of the American Civil Rights movement when its leaders turned an eye toward environmental injustices. Civil rights leaders recognized that Black people were disproportionately affected by pollution but were never included in the modern environmentalism agenda [51]. Thus, the environmental justice movement was not an offshoot of the environmental movement but rather a response to how White and exclusionary mainstream environmentalism was at the time.

This history can in part explain why, outside of the environmental justice context, the term “racism” is scarcely mentioned in prominent environmental health journals and few studies in these journals have featured BIPOC. The studies that have featured majority-populations of color have been impactful. Rauh and colleagues’ longitudinal studies documenting the adverse neurological impacts of prenatal exposure to toxic chlorpyrifos among low-income Black and Dominican children received widespread attention and have contributed to regulatory action [52, 53]; in 2020 a ban on sales in both California and the E.U. took effect [54, 55]. This BIPOC cohort is one of the few regularly accessed in high impact environmental epidemiology journals.

### Male infertility as an illustration

The impact of environmental exposures on male infertility is just one example of how structural racism can be found at the juncture of epidemiology and environmental health, and how it perpetuates health disparities. Human semen, in addition to being an indicator of fertility potential, is an informative marker of general health, and poor semen quality is related to comorbidity and mortality risk [56–58]. Major multi-decade declines in Western sperm counts [59] have received worldwide attention. However almost all that is known about population sperm health comes from White men only and a paucity of U.S. and other Western data exist on the basic semen parameters of men of color [60, 61]. The few studies examining this question have reported lower semen values among Black men compared to other racial and ethnic groups [60, 62–65]. Most of these studies have not ventured to hypothesize why such differences exist, while others have offered cursory genetic or cultural explanations [60, 64]. Environmental toxins including lead, pesticides, air pollution and plasticizers [66–69] are suspected of affecting global sperm declines, yet very few men of color are recruited into reproductive health studies despite having higher body burdens of these chemicals [35] brought upon by decades of environmental racism. Such omissions foster a vast knowledge gap in which researchers know very little about the effect of contaminants on sperm profiles, and by extension the fertility experience of men of color.

The dearth of knowledge about the sperm health of men of color is the consequence of structural factors. In setting the current reference parameters for categorically “normal” sperm values, the World Health Organization (WHO) evaluated studies of men with proven fertility from Australia, France, Denmark, Finland, Scotland, Norway and the United States (*n* = 1953 [70]). Only one of these studies, conducted in the United States, reported on racial variation in its study population, mentioning that fertile non-White men had lower semen volume than fertile White men [71]. Although the WHO acknowledged that the studies it evaluated overlooked populations of color [70], the ramifications are important: current global standards for determining normal versus abnormal semen parameters are based almost entirely on White men and have been used for more than a decade in assisted reproduction treatment clinics worldwide.

Many sperm health studies sample from In Vitro Fertilization (IVF) clinics that are more likely to see men from higher socioeconomic backgrounds who also tend to be White. This phenomenon of “stratified reproduction” has hidden the infertility experiences of BIPOC, including of Black, Latinx, and Arab Americans [72]. The rare efforts that have been made to recruit racially diverse and community-based populations [65, 71, 73, 74] need to be replicated so that study findings are more generalizable to diverse populations, which can increase opportunities to improve treatments and health outcomes for currently underserved populations [75, 76].

Though they are rarely included in fertility studies, BIPOC communities are actively targeted for HIV and other sexually transmitted disease (STD) studies. While the HIV mortality rate among Black men is nine times that of White men [18], it isn’t known whether BIPOC men are at higher risk for infertility or non-infectious reproductive diseases. The disproportionate focus on recruiting BIPOC men as HIV/AIDS research participants while not including them in fertility studies is stigmatizing and demonstrates structural biases in the research process that will require purposeful efforts to deconstruct. The term “reproductive health” should not convey “STD/HIV” for populations of color and “fertility” for White populations.

The call for greater inclusion of BIPOC in male reproductive health studies comes while also acknowledging the long history of exploitation of these communities in medical research. The U.S. Public Health Service’s 40 year Tuskegee Syphilis Study conducted on Black American sharecroppers and the Indian Health Service’s efforts in the 1960s and 70s to sterilize Native Americans are two historical examples in a long legacy of egregious research misconduct [77, 78]. Mistrust of clinical research among marginalized communities of color is well-documented, manifesting as beliefs that it may be conducted without knowledge or consent, on unwilling participants, or that it may increase risk of harm from medical treatments and procedures [79–82]. One study found that Black American mistrust in physicians and clinical research remained significant after accounting for sociodemographic variables such as income or education [79]. The origins of this mistrust are well documented [83], and are a major barrier to health research participation for BIPOC communities [84] largely due to centuries in which the biomedical enterprise took advantage of powerless people who were often of color and poor.

Male infertility is simply one illustration among many  health disparities that are poorly understood because of structural racism in epidemiology and environmental health. There are many examples of how environmental racism likewise affects populations of women of color, including Black women [85–87]. The environmental impacts on sperm health are highlighted here because of the years we have spent studying this issue. The next sections unpack how structural racism is manifest and how discriminatory beliefs, implicit or explicit, have framed public health.

### Manifestation of the problem - White perspectives are centered

Structural racism emanates from systems that are homogenous and poorly diversified. In epidemiology, White identity is normative [88] while “the minority” is an afterthought. White normativity is so engrained in public health research that White is reflexively selected as the referent group in statistical analysis without consideration of the purpose or effect of the choice. This is not entirely surprising, given that students and faculty of color are woefully underrepresented in public health training [89], and accredited public health degrees have minimal requirements to educate about racism and the social determinants of health [90]. Several popular introductory epidemiology texts devote zero to only a few pages to discussing race or the social determinants of health [91, 92], and the historical systems of oppression that underpin these determinants are often missing entirely.

White normativity results in a research culture in which the BIPOC American health experience is poorly understood and marginalized BIPOC populations are systematically neglected [88]. This neglect extends to research teams. A 2012 National Institutes of Health (NIH) working group report revealed that Black, Hispanic or Latino, AI/AN, and Native Hawaiians and other Pacific Islanders made up 1.1, 3.5, 0.2, and 0.1% of NIH principal investigators on research project grants, despite encompassing 12.6, 16.3, 0.9 and 0.2% of the U.S. population, respectively [93, 94]. NIH also found that four and 10 % fewer R01 applications were funded for Asian and Black applicants, respectively (*p* < 0.001), than for White applicants when all qualifications were equal. Trends were similar, though not as stark, for Hispanic applicants [95]. The NIH attributed the Black and White applicant award gap in part to reviewers disfavoring research at the community and population level [96]. International ethical standards for scholarly publication devote one sentence to diversity and inclusion [97]. White professionals serve as the primary gatekeepers to publication [98, 99], the leading currency by which scholarly success is evaluated.

These underpinnings of White as normative contribute to the continued lack of inclusion of populations of color in large cohorts [94], the absence of their control in what and how research questions are posed, and the major deficits in knowledge about racial health disparities. Of the more than 10,000 cancer clinical trials funded by the National Cancer Institute in 2013, less than 2% specifically focused on minority patients despite higher cancer prevalence in these populations [75]. Less than 5% of all NIH-funded studies on respiratory disease reported the inclusion of minority participants [100]. Despite multifactorial barriers to participation in medical research, including mistrust [78, 84], there is evidence that BIPOC express a willingness to participate in health studies at rates similar to non-Hispanic Whites [101]. Thus, the ultimate responsibility for persistent and vast knowledge gaps lies with researchers.

The lack of stable funding also presents a major barrier to enduring health disparity research. The National Institute of Environmental Health Sciences (NIEHS) directed the Division of Extramural Research and Training to provide targeted EJ funding opportunities, primarily through R01, R25, and U45 grants, with a total of 155 grants awarded between 1994 to 2012 [102]. While NIEHS’ EJ funding has contributed substantially to the understanding of disproportionate exposures experienced by communities of color [1, 103–105], funding and science agency priorities are subject to the political preferences of each federal administration [106] such that the investments in EJ research and its promotion have declined in recent years. The interests of marginalized communities are insufficiently integrated into mainstream science and academia and are continuously at risk of being curtailed by unsympathetic administrations [106].

### Underlying factors – lack of focus on structural racism

White normativity is not the only structural barrier impeding a more comprehensive understanding of racial health disparities. In modern epidemiology, researchers utilize scientific examination and quantification of biological and environmental phenomena to explain specific disease states in individuals [107]. Although the race variable is often used in public health research, many researchers and journals fail or refuse to interrogate racism as a critical driver of health inequities [108–110] or address its effects on their findings [111]. The hyper positivist tradition that avoids the social dimensions of health has stifled epidemiology’s ability to improve population health for all, and it all too often relies myopically on empirical methods that reduce race to a data point [1, 112]. Consequently, differences in health outcomes with discernible racial associations have been characterized, intentionally or through neglect, as biological distinctiveness [11].

Attempts in epidemiology to account for race statistically often do so ineffectively [113–115]. A 2004 review of all articles published in top epidemiological journals from 1996 to 1999 (*n* = 1198) found that 919 articles (77%) referred to race or ethnicity, but in studies identifying a race variable (*n* = 787), 57% did not state its purpose and 49% failed to state a statistical rationale for its inclusion [113]. Causal explanations for racial disparities are rarely examined [116]. Disparate exposure to environmental toxins is often discussed without being contextualized with respect to discriminatory origins, such as legally sanctioned segregated housing practices [117, 118]. Other frameworks that have attempted to capture the effects of lifetime exposures on health, such as the exposome [119] and socio exposome [120], incorporate exposures that are byproducts of structural racism, yet rarely mention racism as a driver [121]. For decades numerous researchers have challenged epidemiology to reassess how race and racism are conceptualized, measured, and explained [4–6, 114, 122, 123]. Although methods seeking the causal link between race and health and disease are evolving [124, 125], the calls to name racism have not been realized [9].

Using biology alone to explain health disparities is an example of biological determinism [126], used historically to justify assertions of the mental inferiority of women [127, 128], the intellectual inferiority of immigrants [129], and the genetic inferiority of non-Whites [130] and economically impoverished people [131]. When public health researchers and practitioners reduce disparate health outcomes between White and BIPOC populations to biology without interpreting why or how the social construction of race drives these differences, the implication is that BIPOC are biologically inferior. Whether a result of flawed methodology or conceptual barriers in the field that discourage comprehensive investigation [132], these shallow characterizations are detrimental. Many social scientists have demonstrated that race is a social construct that was formed to uphold White supremacy and justify the subjugation of non-White populations through colonialism and enslavement [108, 133]. Racial categories were based on superficial phenotypic differences, and “race and racism recruited biology” to explain these differences [133]. Biological determinism has been refuted by modern scientists [126] but its pervasiveness in epidemiology is an important example of structural racism in public health. A limited focus on individual risk factors allows public health to justify the circumstances of those marginalized by inequity as “necessary and beyond our control” and disengages the field from social policy solutions aimed at advancing equity [131].

With researchers entrenched in a positivist approach [134] and lacking adequate training to analyze racism as a “cause of causes” [135], racism has been narrowly framed as an individual attribute rather than a structural barrier to health [112]. Analysis of racism as a health predictor is becoming more frequent but still often limited to “interpersonal acts of discrimination” [110] and discussions of structural racism are rare [9]. Hardeman’s 2018 systematic review of the 249 highest impact journals spanning six public health categories found 25 U.S. articles published between 2002 and 2015 that mentioned structural forms of racism in the title/abstract [9]. Rare attempts to elucidate structural racism as a root cause of environmental health disparities [136] have at times led to backlash [137], resulting in authors needing to divert their scholarship to address detractors and defend their work [138]. The bar for legitimizing the study of racism in health research remains simply too high.

### Ramifications

Exclusion of BIPOC researchers and participants and lack of depth in considering race and racism in environmental health and epidemiology have harmful consequences on environmental health protections for all. Analyses supporting biological bases for racial health disparities still emerge in top journals [109] even where an environmental basis might be more probable [139, 140]. Theories that poor health results from genetic differences have eugenic undertones and ramifications beyond only population health. For example, among White medical students and residents (*n* = 222), approximately 50% believed at least one false statement that Blacks were biologically different from Whites with respect to aging, nerve ending sensitivity, skin thickness, fertility, or other measures [8, 141], which affected their ability to assign proper pain control treatments to Black patients in a hypothetical scenario [141]. These and other forms of bias, stereotypes, and prejudice in health care settings influence the experiences and care of patients of color and contribute, along with a myriad of other structural factors, to existing racial and ethnic health disparities [3]. Using biological explanations for racial differences based on the social construct of race is illogical, irresponsible, and misplaced in scientific discourse [109].

Because environmental health research informs health policy, the structures of racism also affect health policy translation and environmental health policy, which have long been hampered by overt and implicit racial injustices [142, 143]. Failure to critically evaluate racism in environmental health research can result in deficient federal and state environmental protections for communities who are disproportionately affected [143, 144]. Recent public health disasters such as the Flint, Michigan lead contamination crisis demonstrate the widespread impacts of regulatory inaction on environmental hazards largely affecting poor and BIPOC communities [144, 145].

## Solutions

Redressing structural racism in environmental epidemiology will require implementation of systemic remedies at all levels of education and research. A variety of proposed solutions are considered herein. Implementation of these or similar countermeasures have the potential to shape the field into one that embraces antiracism as a cornerstone. While not exhaustive, this short list provides a practical starting point toward impactful change.

### Acknowledge racism in public health research

As a matter of course, research in environmental health and epidemiology should address the manner in which pervasive, historical and entrenched discrimination affects multiple pathways across the life course and the lived realities of marginalized populations [146]. This requires a willingness to include racism as a factor in health outcomes and clearly recognize it in publications [4–6, 122] even where it cannot be precisely explained or quantified [147]. Specifically in environmental health, structural racism, already established as a determinant of health [122, 148], should be classified as an exposure.

Beyond simply interrogating racism, researchers should consider and name the way additional systems of oppression intersect to impact health outcomes. Intersectionality, which describes how the intersection of social identities at the micro level, such as race, class, gender, and disability interact with the macro-level structures of poverty, racism and sexism [149], is another prism to evaluate systems of oppression. Incorporating intersectionality into existing environmental health frameworks, like the exposome, to evaluate the impacts of exposures across the lifespan promises to enhance the field’s understanding of how systems of oppression cause health inequities [150]. Innovative work has focused on intersectionality and the exposome in the context of women’s reproductive health [150], and more work is needed in male reproductive health and in other health issues that affect disadvantaged populations at the intersections of race, gender, and class.

### Include affected communities in decision-making

It is imperative that BIPOC scholars are in leadership positions such as service on scientific advisory boards, grant review panels, journal editorial boards and tenured professorships. BIPOC communities bear the legacy of structural environmental racism and are leading many efforts to rectify health disparities, yet significant representation is notably absent from the upper echelons of academia. A scholarship pipeline must be created to promote researchers from these communities. “Diversity and inclusion” statements must be more than just symbolic; they must represent a recruitment mindset that the inclusion of those with a vast array of lived experiences enriches an organization and its scholarship. One concrete solution to hold institutions accountable is to call for the publication of their leadership demographic data, which can ensure institutions recognize and reconcile existing inequities. Organizations should publicly commit to bold hiring targets that prioritize BIPOC inclusion into leadership spaces and create an inclusive rather than exclusive environment that encourages joint learning.

### Develop requirements and standards for discussing “race” in research

Funding sources and journals should outline requirements on how race and ethnicity can be used to guide researchers in experimental design, data analysis, and manuscript preparation. Manuscripts that evaluate racial/ethnic differences or health inequities should explicitly define how race was measured and specify the rationale for including it in the study design. This solution will aid in removing the harmful use of race to explain biological differences [151]. Guidelines such as these have been adopted by the journal *Psychology of Addictive Behaviors*, which requires empirical manuscripts to report on sex/gender and race/ethnicity of the included samples [152]. Their guidelines specify that “examination of race and ethnicity should not be reified as a biological factor and authors should incorporate and explicitly discuss how race and ethnicity may be proxy measures for structural racism” [152]. To be effective, accountability for requirements such as these should apply to grant and manuscript authors and to reviewers [109]. Importantly, researchers need to rethink current statistical modeling approaches that reflexively assign White race as the reference value, a research method that distills White normativity, shifting the focus of research from the inherent advantages of Whiteness to perceived deficiencies in BIPOC communities and inviting an opportunity to amplify existing structural racism rather than elucidating and dismantling it.

### Embrace a more holistic approach to analysis

Researchers need to incorporate structural racism as part of the exposome and should consider other frameworks, such as Life Course Health Development [153] which Gee and colleagues [154, 155] have used to highlight the effect of racism and discrimination on health across life stages. Environmental health research is painstaking in evaluating the effect of chemical exposures at various life stages of development and critical windows of exposure. As exposure to a specific chemical has differential impacts on an individual’s health depending on life stage [156, 157], racism can be considered similarly, as affecting health differently depending on developmental stage [154]. A comprehensive approach needs enlightened research perspectives. This requires moving beyond the strict disciplinary boundaries epidemiologists often uphold, and creating multidisciplinary research teams that include experts from different social science disciplines such as anthropology, demography, geography, psychology, and sociology.

### Partner with community members to conduct research

Community-based participatory research (CBPR) strives to empower study participants by giving them a say in how and what research is conducted [158–160]. Use of CBPR methods to build BIPOC cohorts [1, 136] is crucial but remains underutilized in environmental health research. CBPR includes qualitative methods and reliance on community expertise to inform researchers, empower communities, and build trust [1, 136]; it deserves prioritization and widespread endorsement as a leading environmental health framework. Through CBPR, researchers are challenged not only to recognize health inequities associated with social, economic, and environmental racism but also to work with affected populations to create actionable solutions [158]. For instance, male reproductive health researchers could partner with community organizations to design studies that not only include more diverse cohorts outside the traditional IVF setting, but seek a better understanding of the fertility views and needs of that community. National reports and funding initiatives have called for a more comprehensive and integrated approach to conducting research by actively involving marginalized communities [161, 162], and it is past time for environmental health practitioners to incorporate these measures to dismantle structural racism. Institutions should encourage and incentivize collaboration between researchers and social scientists versed in CBPR who can facilitate healthy relationships with community groups.

Researchers should also prioritize the needs of participants so that participants do not feel exploited and information is exchanged to benefit all stakeholders, as trust among researchers and participants is paramount. A key feature of participatory research is that it preserves the expertise of community members and uses it to advance research priorities within vulnerable and marginalized communities.

### Improve training for researchers and students

To dismantle racist structures, public health professionals need the ability to identify and analyze the impact of racism. Antiracism literacy must be a foundational skill taught throughout public health education [122]. The Council on Education for Public Health (CEPH)'s foundation competencies should demand not just a narrow assessment identifying racism as a factor impacting health equity, but a curriculum informed by antiracism and a diverse body of educators. Public health institutions must ensure that their built-in systems do not perpetuate racism, their teaching materials encompass BIPOC communities, and practices such as “cluster hiring” are implemented to effectively and strategically diversify faculties [163]. Public health education will also benefit from adopting intersectionality as a critical praxis.

Training students and researchers in antiracist literacy may be challenging, but completing courses is a viable approach [164] and competency should be regularly maintained as is required with other competencies (e.g., health information privacy, laboratory safety, sexual harassment, etc.). A multitude of validated racial and cultural literacy self-assessment tools are available [165, 166] as are an abundance of antiracism resources for students and researchers to take in outside of the classroom [167]. These issues are not new, and a body of literature exists educating practitioners about structural racism [108, 168, 169]. Antiracism learning in environmental health should be supported as a model of lifelong professional development.

## Conclusion

The core of environmental health lies in eliminating environmental burdens for all, a legacy of which exists in BIPOC communities. Environmental health is ideally positioned to lead in improving analysis of structural racism as a barrier to health equity. But to do so, the field needs to also examine the way its structures may contribute to inequities, and demonstrate an active willingness to change. The impact of these structures is not unique to environmental health and epidemiology but they limit advances in scientific understanding of environmental components of health disparities, including those related to male reproductive health. Fortunately, the tools and strategies that scholars have long offered provide approaches to account for race, place, and the structural racist forces that have contributed to decades of population health disparities and knowledge gaps. It is past time for researchers to embrace these approaches, and the field of environmental health is uniquely positioned to advance them.

## Data Availability

Not applicable.

## Abbreviations

AI/AN: American Indian/Alaska Native
COVID-19: Coronavirus infectious disease 2019
BIPOC: Black, Indigenous, and people of color
EJ: Environmental Justice
CBPR: Community-Based Participatory Research
WHO: World Health Organization
IVF: In-Vitro Fertilization
HIV: Human Immunodeficiency Virus
STD: Sexually transmitted disease
AIDS: Acquired Immunodeficiency Syndrome
NIEHS: National Institute of Environmental Health Sciences
